# Arabidopsis mutants may represent recombinant introgression lines

**DOI:** 10.1186/s13104-018-3326-5

**Published:** 2018-04-03

**Authors:** Narendra Singh Yadav, Janardan Khadka, Gideon Grafi

**Affiliations:** French Associates Institute for Agriculture and Biotechnology of Drylands, Jacob Blaustein Institutes for Desert Research, Ben-Gurion University of the Negev, 84990 Midreshet Ben-Gurion, Israel

**Keywords:** *Arabidopsis thaliana*, Ler ecotype, Columbia ecotype, Evelknievel retroelement, Tag1 transposable element, *cmt3*, *kyp2*, *ago4*, *ddm1*, Recombinant introgression lines, Backcrossing

## Abstract

**Objectives:**

It is a common practice in *Arabidopsis* to transfer a mutation generated in one genetic background to other genetic background via crossing. However, the drawback of this methodology is unavoidable presence of genomic fragments from the donor parent being often replacing desirable genomic fragments of the recurrent parent. Here, we highlighted problem of Arabidopsis mutants being recombinant introgression lines that can lead to unreliable and misinterpreted results.

**Results:**

We studied the regulation of low copy number transposable elements Tag1 and Evelknievel (EK), located at the end of the bottom arm of chromosome 1 and both are present in the *Arabidopsis* Landsberg erecta (Ler) but not in Columbia (Col) ecotype. Using various epigenetic mutants (*cmt3*, *ddm1*, *kyp2*, *ago4*, *rdr2 hen1* etc.), we found that certain mutants in the Ler background are deficient of Tag1 or EK or both and represent recombinant introgression lines whereby chromosomal regions from Col have been recombined into the Ler genome. Our data support a recent proposal calling for formulating standards for authentication of plant lines that are used in plant research. Most important is to verify that a given trait or genomic locus under study is correctly identified, particularly when using mutants generated by crossing.

**Electronic supplementary material:**

The online version of this article (10.1186/s13104-018-3326-5) contains supplementary material, which is available to authorized users.

## Introduction

Contamination and misidentification of cell lines is a common, long-standing problem in medical research calling for establishing proper controls and standards for cell culture authentication [1]. Obviously, studies that are conducted with misidentified cell lines are deceptive, misconceived by the scientific community adding disinformation to the literature that might affect future studies [2]. In a recent letter, Bergelson et al. [3] raised a concern regarding the identity of the plant genetic material used by plant biologists including transgenic lines, mutants, or accessions claiming that plant lines “may not be what they are supposed to be”. The authors suggested formulating standards for validation of genetic stocks to avoid contamination and misidentification of genetic material used in plant research. Indeed, a recent report demonstrated SNP match as an efficient tool for genotyping Arabidopsis stock collections [4]. Here we highlight the necessity for developing standards for genotyping and identification of plant material by describing a special case whereby mutant lines in the *Arabidopsis thaliana* Landsberg erecta (Ler) genetic background appear to be recombinant introgression lines between Ler and Columbia (Col) ecotypes where desirable genomic regions of Ler were replaced by the corresponding, yet undesirable genomic regions of Col ecotype.

## Main text

### Materials and methods

#### Plant materials

We studied wild type Col and Ler, as well as mutants in the Ler background, namely, *ddm1* (Ler background CSHL-GT24941), *cmt3*–*7* (CS6365, provided by Autran) and *kyp2* (CS6367, provided by Autran), *hen1* (provided by Mlotshwa, V. Vance lab) and *rdr2 hen1* double mutant (Bin Yu lab). In addition, five *ago4*-*1* lines (Ler background) obtained from various labs were analyzed including *ago4*-*1*a (Zilberman lab, University of California, Berkeley, USA; ABRC CS6364), *ago4*-*1*b and *ago4*-*1*c (Daphne Autran lab, IRD, University of Montpllier, France; ABRC CS6364), *ago4*-*1*d (Judith Bender lab, Brown University, USA; ABRC CS6364) and *ago4*-*1*e (Caroline Dean, John Innes Centre, UK). All *Arabidopsis thaliana* lines, were grown in a controlled growth room under long day photoperiod (16 h light and 8 h dark, light intensity 200 μmol photons m^−2^ s^−1^) at 22 °C ± 2 and 70% humidity.

#### DNA isolation and PCR analysis

DNA was extracted from wild type and mutant leaves using Genomic DNA Mini kit (Cat. No. GP100, Geneaid, Taiwan). This DNA was subjected to PCR to amplify the Tag1, Evelknievel (EK), indel-1, indel-7, indel-9 and nga225 (for primer sequences see Additional file 1). PCR conditions were 95 °C, 5 min; 30–40 cycles of 95 °C, 30 s; 60 °C, 30 s; 72 °C, 30 s; followed by 72 °C, 5 min. PCR products were resolved on 1.5% agarose (SeaKem LE AGAROSE Cat. No. 50004, Lonza, USA) gel stained with ethidium bromide. The PCR analysis repeated at least three times.

### Results and discussion

**Fig. 1:**
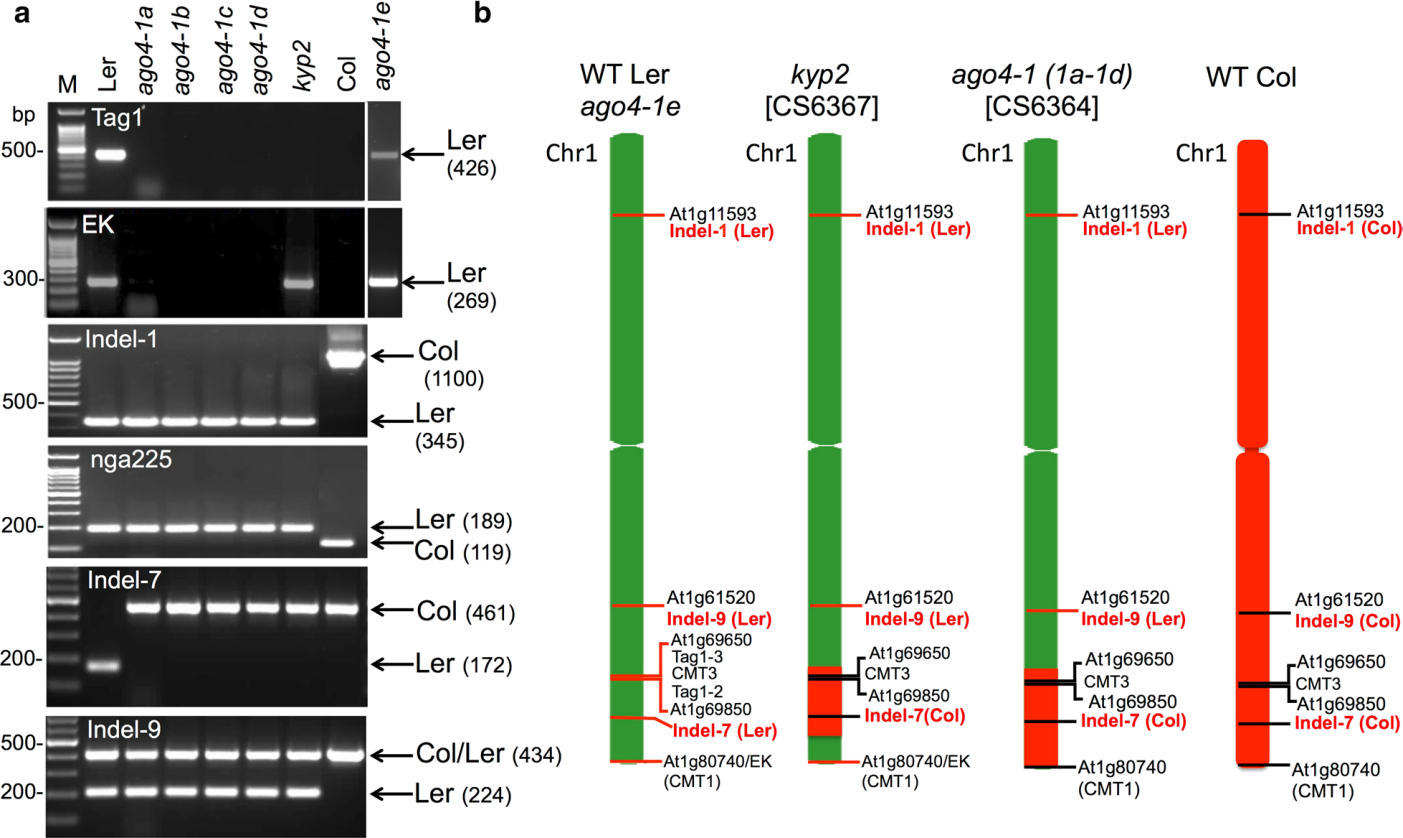
Genotyping of *kyp2* and *ago4*-*1* mutants. **a** PCR analysis of the indicated markers known to distinguish Ler from Col. Five *ago4*-*1* lines obtained from various labs (named as a, b, c, d and e) and *kyp2* mutant were analyzed. Four *ago4-1 lines* (*a*–*d*) do not contain both Tag1 and EK, while *kyp2* mutant contains EK only. Note that only *ago4*-*1e* possesses the authentic chromosome 1 bottom arm of Ler containing both Tag1 and EK. **b** Schematic representation demonstrating the recombinant nature of *kyp2* and *ago4*-*1* mutant lines. Red boxes represent chromosomal fragments related to Col genotype. The TAIR Chromosome Map Tool was used to make positional genome map of these polymorphic markers and the nearest gene locus TAIR id is shown. *EK* Evelknievel retroelement

In an attempt to gain insight into the mechanism(s) by which transposable elements are activated in the course of protoplasting-induced cell dedifferentiation, we have shown previously that the class II, low-copy-number Tag1 transposable elements (TEs), which exist in Ler but not in Columbia (Col) ecotype is activated in dedifferentiating protoplasts and that CMT3 appears to be the major factor controlling their activity via inducing gene body CHG methylation [5]. Two copies of Tag1 elements are situated close to each other at the end of bottom arm of chromosome 1 (between At1g69650 and At1g69850 loci). Since CMT3 and KYP/SUVH4 act together to reinforce silencing of certain TEs [6], we wanted to address the involvement of KYP/SUVH4 in the regulation of Tag1 elements. We obtained *kyp2* mutant in the Ler background (CS6367 or NASC id: 6367) and to our surprise, our analysis revealed that Tag1 elements are not present in this mutant line (Fig. 1a, Tag1 panel) and we assumed that we got a *kyp* mutant line in the Col background by mistake. Furthermore, to reveal the possible involvement of RNA-dependent DNA methylation (RdDM) in silencing of Tag1 elements we obtained five *ago4*-*1* mutants in the Ler background from various labs most of them appear to be related to CS6364 or NASC id 6364. Surprisingly, out of the five, four *ago4*-*1*(a–d) mutants were deficient of the Tag1 transposons (Fig. 1a, Tag1 panel) leading us to assume that these mutants are either in the Col genetic background or that Tag1 elements were eliminated from genome in these mutants. To confirm that the genetic background of *ago4*-*1* and *kyp2/suvh4* mutants is indeed Ler, we used three markers reported previously to distinguish Ler from the Col ecotype including Evelknievel (EK) a copia-like retroelement inserted within the *CMT1* gene (At1g80740), which exist in Ler but not in Col genome and is localized at sub-telomeric region of bottom arm of chromosome 1 [7]. In addition we used microsatellite nga225 [8] and indel-1 marker (NCBI accession no. EU737117) [9]. All markers (Fig. 1a) clearly confirmed that *kyp2/suvh4* mutant is in the Ler background supporting the hypothesis that Tag1 may have been eliminated from genome due to lack of KYP/SUVH4 HMTase. Surprisingly, however, while indel-1 and nga225 confirmed that all *ago4* mutant lines are in the Ler background, Tag1 and EK were absent in *ago4*-*1* mutants (Fig. 1a, EK panel) leading to the erroneous initial conclusion that AGO4 might be required for maintaining low copy number class I (EK) and class II (Tag1) TEs in the *Arabidopsis* genome.

**Fig. 2:**
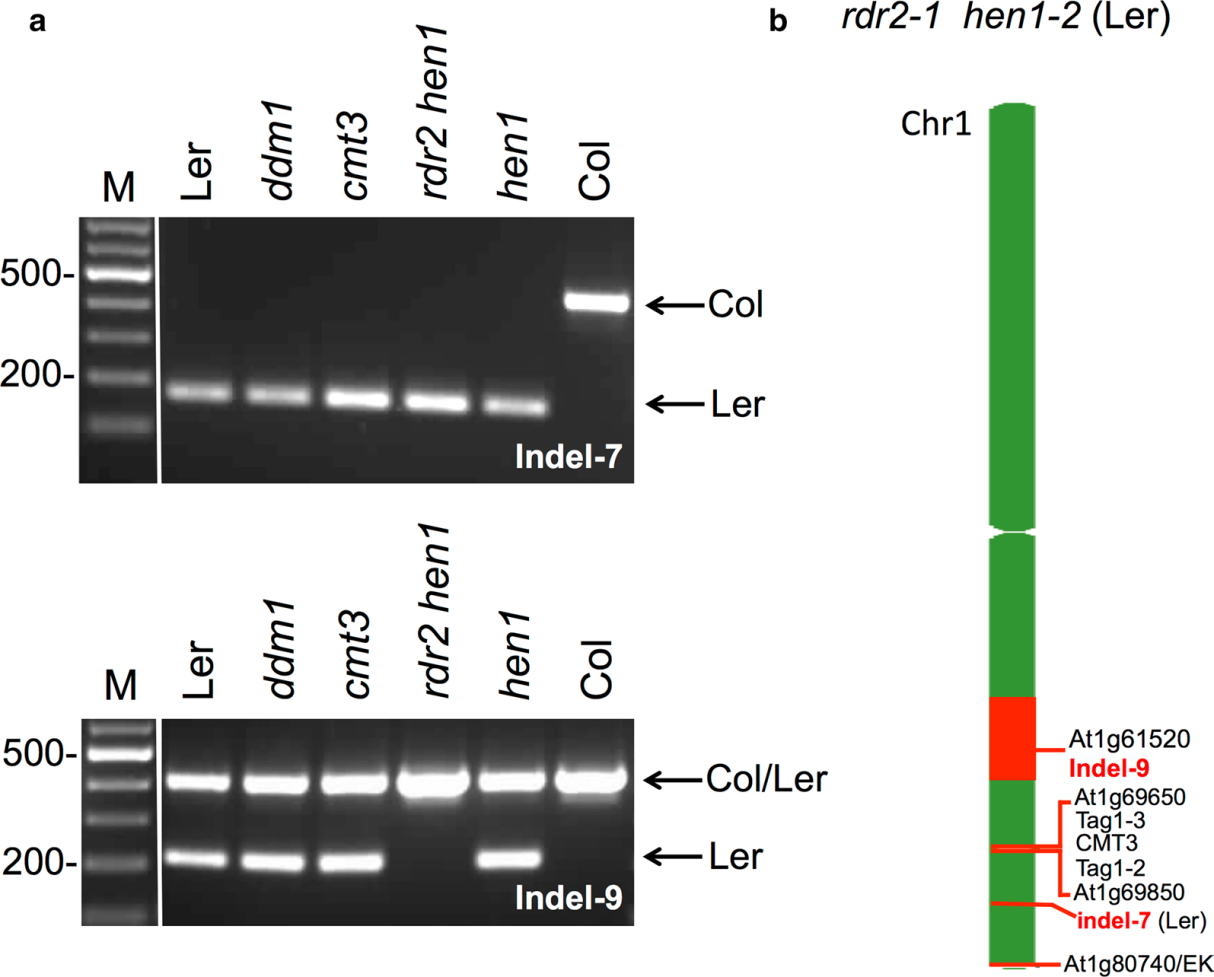
Genotyping of various mutants in the Ler background. **a** PCR analysis of the indel-7 and indel-9 markers in *hen1*, *rdr2 hen1*, *cmt3* and *ddm1*. PCR analysis of wild type Ler and Col was used as a reference. Amplified fragments related to Col or Ler are indicated by arrows. Note that *rdr2 hen1* double mutant displays Ler background with indel-7 marker but Col with indel-9 marker. **b** Schematic representation demonstrating the recombinant nature of *rdr2 hen1* mutant line. Red boxes represent chromosomal fragments related to the Col genotype. The TAIR Chromosome Map Tool was used to make positional genome map of these polymorphic markers and the nearest gene locus TAIR id is shown

We were aware that a common practice in generation of mutants in various *Arabidopsis* accessions is by crossing a mutant line in one genetic background with another accession followed by backcrossing to recover the mutation in the new genotypic background. Indeed, some mutants in the Ler background were actually generated via hybridization of Ler with Col mutants e.g. *rdr2*-*1 hen1*-*2* double mutant [10] while in others, the crossing with Col background has been carried out to map Ler background mutants (e.g. *ago4*-*1 and kyp2*). However, the drawback of this methodology is the unavoidable formation of recombinant introgression lines where Col chromosomal regions are recombined into the Ler genome. Thus, we hypothesized that *ago4*-*1* and *kyp2* mutants in the Ler background might represent recombinant introgression lines whereby fragments of the bottom arm of chromosome 1 from Col have been recombined into the Ler genome. To assess this possibility, we selected two additional indel markers including indel-7 located at the bottom arm of chromosome 1 between Tag1 and EK elements and indel-9 on bottom arm of chromosome 1 near At1g61590 gene locus (Fig. 1b). The results showed that while indel-9 marker clearly identified *kyp2* and all *ago4*-*1* mutant lines as Ler background, indel-7 marker, which is associated with the chromosomal region under study, was identical to WT Col ecotype supporting the hypothesis that chromosomal fragments derived from the bottom arm of chromosome 1 of Col have been recombined into the Ler genome in *ago4*-*1* (a-d) and *kyp2* mutants (Fig. 1b). Furthermore, in *kyp2* mutant Tag1 marker is absent and indel-7 is of Col origin, but EK is present suggesting that this region containing the Tag1 elements and indel-7 DNA sequence has been replaced with its corresponding chromosomal region from Col ecotype (Fig. 1b). Notably, indel-7 and indel-9 markers revealed that *cmt3*, *ddm1* and *hen1* mutants have the authentic Ler chromosome 1 bottom arm; *rdr2 hen1* double mutant displays Ler background with indel-7 marker, but Col with indel-9 marker (Fig. 2).

**Fig. 3:**
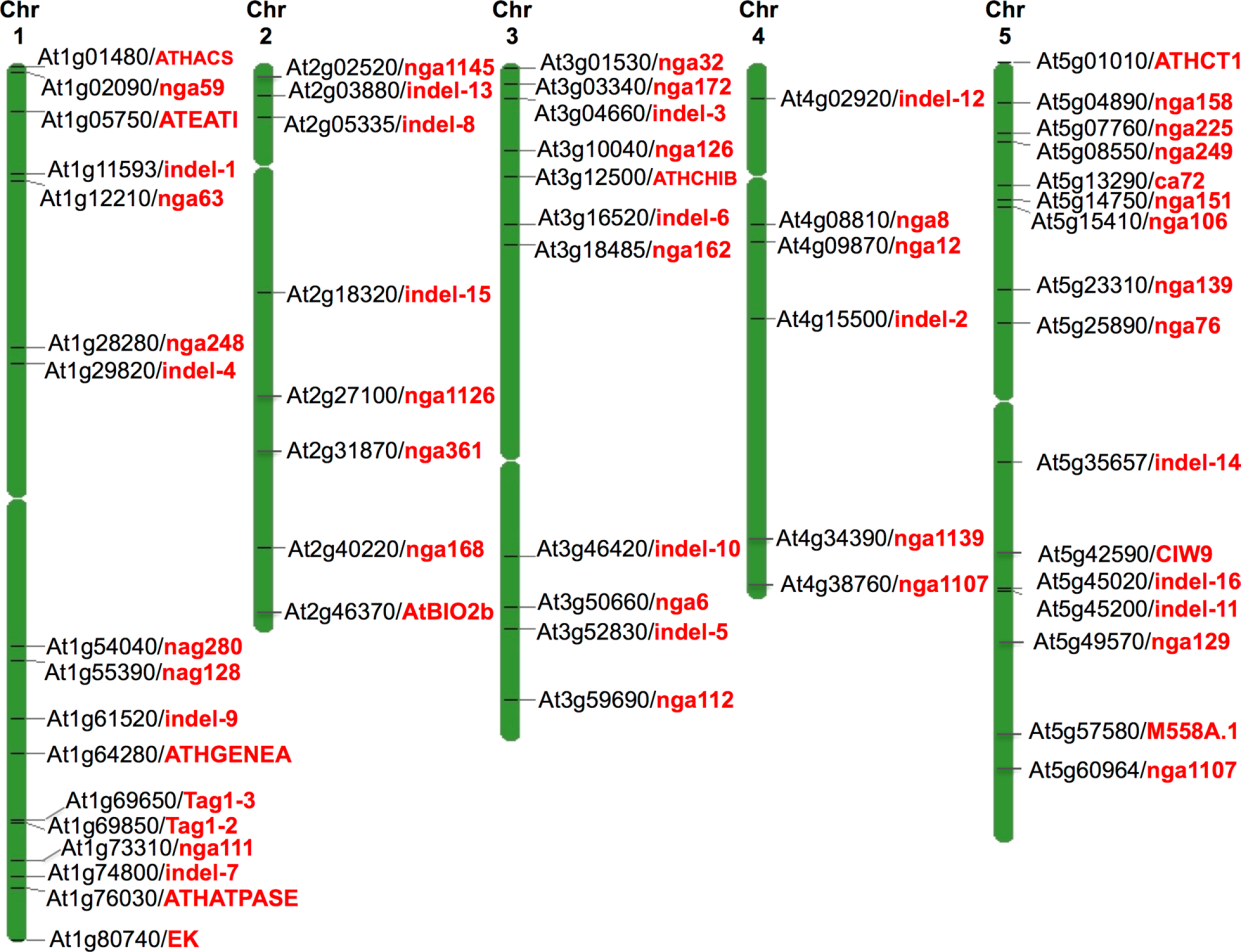
Schematic representation of various polymorphic markers positions on chromosomes to distinguish Arabidopsis Col and Ler ecotype. The 30 microsatellite loci [8], 16 indels loci [9], two Tag1 loci [11], one Evelknievel (EK) locus [7] and some polymorphic markers from TAIR Marker Search (http://www.arabidopsis.org/servlets/Search?type=marker&action=new_search) are shown. The TAIR Chromosome Map Tool was used to make positional genome map of these polymorphic markers and the nearest gene locus TAIR id is shown

Thus as recently suggested [3] it should be made mandatory to verify the identity of plant genetic stocks that are used by plant biologists. Particular attention should be given to plant lines where a mutation in one genetic background is transferred into another background by means of crossing/backcrossing. Validating the genotypic background is possible by using various polymorphic markers that can distinguish between Arabidopsis ecotypes including Microsatellite and indel markers ([7–9, 11]; Fig. 3). More polymorphic markers for genotyping of Arabidopsis strains can be find at TAIR website under ‘TAIR Marker Search’ (http://www.arabidopsis.org/servlets/Search?type=marker&action=new_search). Recently, Pisupati et al. [4] have developed SNP match tool that identifies Arabidopsis strains by matching them to a SNP database (https://arageno.gmi.oeaw.ac.at/). In conclusion, it is important to verify that a given trait or genomic locus under study is correctly identified.

## Limitations

Our study is limited to the genomic locus containing the transposable elements Tag1 and Evelknievel, which are located at the end of the bottom arm of chromosome 1 of *Arabidopsis thaliana* WT Ler ecotype; these TEs are not present in the WT Col ecotype. Thus our study is limited to the analysis of only these TEs; other TEs in other chromosomal regions were not studied. In this study, we have verified various epigenetic mutants for introgression only at the bottom arm of chromosome 1, but we didn’t test the possibility for introgression in other chromosomal loci. We assembled a practical tool of various polymorphic markers covering large part of the Arabidopsis genome that can be used for the assessment of introgression between Arabidopsis ecotypes. The work is also limited to only mutant lines used in this study and derived from crosses between Ler and Col ecotypes.

## Additional file

**Additional file 1.** List of primers used in this study.

## Abbreviations

CMT1: chromomethylase1
CMT3: chromomethylase3
DDM1: decrease in DNA methylation1
Ler: Landsberg erecta
Col: Columbia
AGO4: argonaute4
EK: Evelknievel
KYP/SUVH4: kryptonite/suppressor of variegation homolog4
RdDM: RNA dependent DNA methylation
