# Supplementary material for: Arabidopsis mutants may represent recombinant introgression lines
Source: BMC Res Notes. 2018 Apr 3;11:227. doi: 10.1186/s13104-018-3326-5 (PMC5883871; doi:10.1186/s13104-018-3326-5)
Supplement: Supplementary file 1 — Additional file 1. List of primers used in this study. [file 13104_2018_3326_MOESM1_ESM.docx]

**Additional file 1**

**Arabidopsis mutants may represent recombinant introgression lines**

Narendra Singh Yadav*, Janardan Khadka and Gideon Grafi

French Associates Institute for Agriculture and Biotechnology of Drylands, Jacob Blaustein Institutes for Desert Research, Ben-Gurion University of the Negev, Midreshet Ben Gurion 84990, Israel.

*Corresponding author: [nsyadava2004@gmail.com](mailto:nsyadava2004@gmail.com); [yadav@post.bgu.ac.il](mailto:yadav@post.bgu.ac.il)

**Additional file 1. List of primers used in this study.**

| **Oligo Name** | **Sequence (5' to 3')** | **Expected size(bp)**  **Col** | **Expected size (bp)**  **Ler** |
| --- | --- | --- | --- |
| Tag1 | TTGATATGGCTTTAACTTGCAGC | Absent | 426 |
|  | GTAGTCAACAGGATCATATGATC |  |  |
| EK1 | TTCGCAACTGCTCGATATTCT | Absent | 269 |
|  | CACTGTCCAACCGATCTTCAT |  |  |
| Indel-1 | GCAAATGGAGTTGGAAGATCA | 1100 | 345 |
|  | GGTTGGCCAATTGTTAATGTCT |  |  |
| Indel-7 | TCCAATGGACACAAACAAGACT | 461 | 172 |
|  | CAAGTCTAGCCTTCAAATTTCC |  |  |
| Indel-9 | GAGACTGCTCTTCCTTGGTT | 434 | 434  224 |
|  | CTGGTCAAGACGTTGTTGTTGA |  |  |
| nga225 | GAAATCCAAATCCCAGAGAGG | 119 | 189 |
|  | TCTCCCCACTAGTTTTGTGTCC |  |  |
